# ﻿Comparative analysis of mitogenomes in six *Scolopendra* species (Chilopoda, Scolopendromorpha): insights into rare genetic rearrangements and phylogeny

**DOI:** 10.3897/zookeys.1248.159578

**Published:** 2025-08-11

**Authors:** Mengcheng Wan, Yiwen Yang, Jiachen Wang, Gaoji Zhang, Hongyi Liu, Cuiqing Gao

**Affiliations:** 1 Co-Innovation Center for Sustainable Forestry in Southern China, College of Forestry and Grassland, Nanjing Forestry University, Nanjing, Jiangsu 210037, China Nanjing Forestry University Nanjing China; 2 College of Life Sciences, Nanjing Forestry University, Nanjing 210037, China Nanjing Forestry University Nanjing China

**Keywords:** Centipede, gene duplication, gene rearrangement, mitochondrial genome, mitogenomic structure, phylogeny, Scolopendridae, Scolopendrinae

## Abstract

Six complete mitochondrial genomes from species within *Scolopendra* (Scolopendromorpha) (*S.alcyona*, *S.calcarata*, *S.cataracta*, *S.lufengia*, *S.mazbii*, and *S.multidens*) were analyzed. The mitochondrial genomes ranged in size from 14,422 bp to 15,458 bp, with A+T content varying between 67.00% and 74.53%. All PCGs and rRNA were successfully identified, though tRNAs exhibited widespread loss. Additionally, supplementary control region sequences were identified in three species. Beyond sequence data, the arrangement of mitochondrial genes can provide additional phylogenetically relevant information. The mitogenome arrangements in *S.alcyona*, *S.cataracta*, *S.lufengia*, and *S.mazbii* are consistent with the inferred ancestral arrangement in Myriapoda, but *Scolopendramultidens* had a duplication of tRNA and *S.calcarata* underwent a significant gene rearrangement. The Tandem Duplication/Random Loss model and tRNA mispriming mode were determined as most likely explanations for the observed gene rearrangements. Phylogenetic trees were reconstructed using the six newly sequenced mitogenomes and published mitochondrial data from other Chilopoda species. The results revealed two major monophyletic clades within *Scolopendra*: one comprising *S.alcyona*, *S.dehaani*, *S.cataracta*, *S.lufengia*, *S.morsitans*, *S.multidens*, and *S.mutilans*, and the other uniting *S.mazbii* and *S.calcarata*. Notably, *S.subspinipes* unexpectedly clustered with *Scolopocryptops* sp., challenging the monophyly of *Scolopendra* and highlighting the need for revised taxonomic evaluations. These results enhance our understanding of gene rearrangements and evolutionary dynamics in Scolopendromorpha, offering critical insights into their phylogenetic relationships.

## ﻿Introduction

The mitogenome of eumetazoans typically consists of 13 protein-coding genes (PCGs), two ribosomal RNA genes (rRNAs), and 22 transfer RNA genes (tRNAs), along with a non-coding region known as the control region (CR) (Boore 1999). Mitochondrial DNA is widely utilized as a valuable molecular marker in evolutionary studies due to its compact genome size, straightforward structure, maternal inheritance pattern, conserved gene order, moderate mutation rate, and ease of data acquisition (Chipman et al. 2014; Sterling-Montealegre and Prada 2024).

The organization of metazoan mitogenomes can exhibit a relatively stable pattern, with certain arrangements having been preserved for hundreds of millions of years (Boore 1999). While mitochondrial gene order was once thought to be highly conserved, frequent rearrangements within the mitogenome have been observed in arthropods (Lavrov et al. 2002; Zhao et al. 2022), occurring at a significantly higher frequency compared to gene rearrangement events in vertebrates (Ruan et al. 2020; Xu et al. 2021). This variability in gene order generally affects tRNAs rather than PCGs and rRNAs (Shtolz and Mishmar 2023).

Several hypotheses have been proposed to explain gene rearrangements in animal mitochondrial genomes, including the Tandem Duplication/Random Loss (TDRL) model (Moritz and Brown 1987), the Tandem Duplication/Non-Random Loss (TDNL) model (Lavrov et al. 2002), the Recombination model (Lunt and Hyman 1997), and the tRNA mispriming model (Cantatore et al. 1987). Among these, the TDNL and tRNA mispriming models result in distinct rearrangement patterns, while the Recombination model, characterized by gene inversions and applicable mainly to small gene block exchanges, is relatively rare in mitochondrial genomes. One of the most widely accepted hypotheses is the TDRL model, which posits that rearranged gene orders arise through tandem duplications followed by random deletions of certain duplications (Lado et al. 2019). The TDRL model is a prevalent mechanism for significant mitochondrial gene rearrangements and tRNA gene eliminations (Jühling et al. 2012). Mitochondrial gene order rearrangements are considered unique and rare events that are unlikely to occur independently in separate evolutionary lineages due to convergence (Boore 1999), making them powerful phylogenetic markers (Shao and Barker 2003). With recent advancements in next-generation sequencing technologies, which have reduced the cost and complexity of DNA sequencing, complete mitochondrial genomes are increasingly used in phylogenetic analyses (Sun et al. 2019).

Chilopoda (Latreille, 1817), one of the four major lineages within the subphylum Myriapoda, includes five extant orders (Scutigeromorpha, Geophilomorpha, Lithobiomorpha, Scolopendromorpha and Craterostigmomorpha) and approximately 5000 known species. It is the only predatory group among the myriapods (Brewer et al. 2012; Fernández et al. 2014). Scolopendromorpha is one of the two most derived orders within Chilopoda and is either blind, has a single pair of ocelli, or bears a group of four ocelli on each side of the head capsule (Vahtera et al. 2012). Scolopendromorpha comprises the largest and most aggressive predatory centipedes, with around 700 known species found across tropical, subtropical, and temperate regions globally (Edgecombe and Giribet 2006; Edgecombe and Koch 2008). Due to their toxicity, they are a key group in medical and toxicological research (Ma et al. 2015; Ombati et al. 2018). The order Scolopendromorpha comprises three families: Scolopendridae, Cryptopidae, and Scolopocryptopidae. Within the family Scolopendridae, two subfamilies are recognized: Scolopendrinae and Otostigminae. The genus *Scolopendra* Linnaeus, 1758, which belongs to Scolopendrinae, is the only representative of this subfamily found in China. *Scolopendra* is a globally distributed genus with 77 described species, predominantly inhabiting tropical and subtropical regions. In China, nine species (including subspecies) of this genus have been documented (Song 2004; Kang 2018).

Despite its widespread distribution, species identification within the order Scolopendromorpha remains highly challenging, with significant taxonomic controversies persisting. These difficulties arise from morphological variations, overlapping characteristics, and the frequent oversight of ontogenetic, intraspecific, and interspecific variability (Kang et al. 2017). To partially bridge the gaps in the little-published genomes of Scolopendromorpha and better understand its diversity, evolution, and phylogenetic relationships, we sequenced six new complete mitogenomes (*Scolopendraalcyona* Tsukamoto & Shimano, 2021, *Scolopendracalcarata* Porat, 1876, *Scolopendracataracta* Siriwut, Edgecombe & Panha, 2016, *Scolopendralufengia* Kang, Liu, Zeng, Deng, Luo, Chen & Chen, 2017, *Scolopendramazbii* Gravely, 1912, and *Scolopendramultidens* Newport, 1844) and analyzed their mitogenomic structure. We hope that our study will enhance the understanding of Scolopendromorpha biodiversity and expand genetic resources for future comparative studies within this group.

## ﻿Material and methods

### ﻿Sample collection and DNA extraction

The specimens used in this study were sourced from the Qiqiaoweng Pet Market (32.005488°N,118.84162252°E) in Nanjing, Jiangsu Province, China. All specimens were deposited at the Insect Taxonomy Laboratory of Nanjing Forestry University. Genomic DNA was extracted from the legs of each specimen using the FastPure Cell/Tissue DNA Isolation Mini Kit (Vazyme, Nanjing, China) and stored at -80 °C for future analysis.

### ﻿Genome sequencing, assembly, and annotation

Six complete mitogenomes were sequenced using an Illumina Novaseq 6000 sequencing platform (Personalbio, Nanjing, China) from total genomic DNA. The genomic DNA was used to generate an Illumina library with an insert size of 400 bp. The library was generated using the NEB Next® Ultra™ DNA Library Prep Kit for Illumina (New England Biolabs, Beverly, MA, USA) following the manufacturer’s recommendations. All the data were then assembled in Geneious Prime 2024 software, with *Scolopendradehaani* Brandt, 1840 (KY947341.1) as the reference template. The mitogenomes were further assembled and manually revised using DNAstar v. 7.1 (Madison, WI, USA).

Genome annotation was carried out using BLAST (https://www.ncbi.nlm.nih.gov/Structure/cdd/wrpsb.cgi) and the MITOS WebServer (http://mitos.bioinf.uni-leipzig.de/index.py) (Bernt et al. 2013). The 13 PCGs were manually annotated using start codons (ATN, GTG, TTG) and stop codons (T, TA, TAA, TAG). The tRNA genes were identified and their secondary structures were predicted using tRNAScan-SE v. 2.0 (Lowe and Chan 2016) and ARWEN v. 1.2 (Laslett and Canback 2008). Subsequently, the anticodon of missing tRNA was manually identified and its sequence boundaries delineated, with comparative analysis confirming congruence to the target tRNA. Maps of the mitogenomes were constructed using CGView (https://cgview.ca/) (Stothard and Wishart 2005). Base composition, Relative Synonymous Codon Usage (RSCU), pairwise genetic distances (p-distance), and the rates of non-synonymous (Ka) and synonymous (Ks) substitutions were analyzed using MEGA 11 software (Fay and Wu 2003; Kumar et al. 2016). Composition skew values were calculated using the following formulas: AT-skew = (A−T) / (A+T) and GC-skew = (G−C) / (G+C) (Perna and Kocher 1995). All resulting data were visualized and refined using Origin 2024b and Photoshop 2024 software.

### ﻿Phylogenetic analysis

To investigate the phylogenetic distribution of Chilopoda, Bayesian inference (BI) and maximum likelihood (ML) methods were employed to reconstruct two phylogenetic trees based on a concatenated alignment of the 13 PCGs from 17 species. This analysis was performed using the PhyloSuite v. 1.2.3 toolkit (Zhang et al. 2020). Seventeen species from four orders of Chilopoda (Scutigeromorpha, Geophilomorpha, Lithobiomorpha, and Scolopendromorpha) were selected as the ingroup. Only species with available annotation data were included. The outgroup was *Epanerchoduskoreanus* Verhoeff, 1937 a species from Diplopoda, another class within Myriapoda (Table 1). Gene sequences were aligned using MAFFT v. 7.313, and the optimal substitution model and partition scheme were determined with ModelFinder. ML analyses were performed using the GTR+F+R7 model in IQ-TREE. BI analyses using the GTR+I+G4 nucleotide substitution model were performed in MrBayes v. 3.2.6, and four independent runs with four Markov Chain Monte Carlo were conducted, sampling every 1000 generations for 1,000,000 generations. The first 25% of the trees were removed during the burn-in phase, and the remaining trees were used to calculate Bayesian posterior probabilities (Leger et al. 2017). The final trees were visualized and refined using iTOL v. 6 (Letunic and Bork 2024).

**Table 1.** List of the mitogenomes analyzed in this study. The sequences PP816935.1 and PP855997.1–PP856000.1 are newly generated; all others were obtained from NCBI.

Family	Species	Accession no.	Length (bp)
Chilopoda	*Scolopendraalcyona*	PP855997.1	14,422
	*Scolopendracalcarata*	PP855998.1	15,458
	*Scolopendracataracta*	PP856000.1	14,596
	*Scolopendradehaani*	KY947341.1	14,538
	*Scolopendralufengia*	PP816935.1	14,647
	*Scolopendramazbii*	PP855999.1	14,496
	*Scolopendramorsitans*	MW810062.1	14,019
	*Scolopendramultidens*	PP855996.1	14,590
	*Scolopendrasubspinipes*	MN642577.1	14,637
	*Scolopendramutilans*	MN317390.1	15,011
	*Scolopocryptops* sp.	KC200076.1	15,119
	*Scutigeracoleoptrata*	AJ507061.2	14,922
	*Bothropolys* sp.	AY691655.1	15,139
	*Cermatobiuslongicornis*	KC155628.1	16,833
	*Lithobiusforficatus*	AF309492.1	15,692
	*Strigamiamaritima*	KP173664.1	14,983
	*Mecistocephalusmarmoratus*	KX774322.1	15,279
Diplopoda	*Epanerchoduskoreanus*	MT898420.1	15,581

## ﻿Results

### ﻿Genome organization and composition

**Figure 1.**
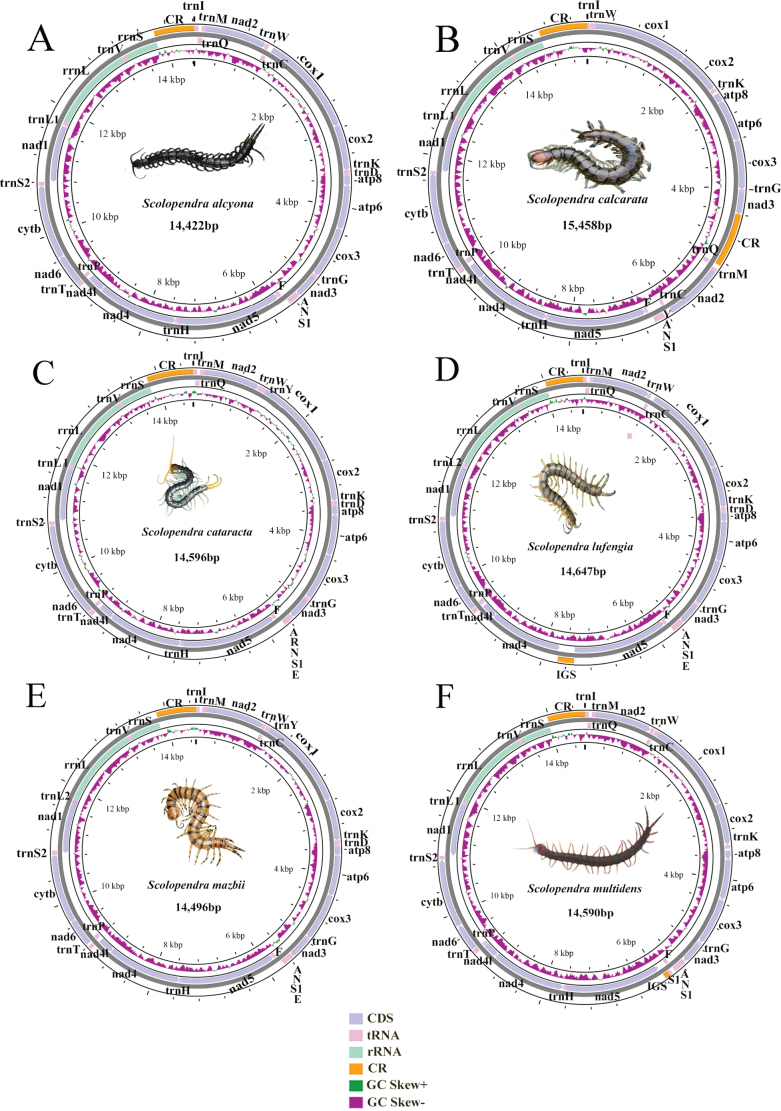
Gene maps of the six newly sequenced mitogenomes. Different gene types are color-coded.

Six complete mitogenomes from the six species of *Scolopendra* (Scolopendromorpha) were obtained. The lengths of *S.alcyona* (14,422 bp), *S.cataracta* (14,596 bp), *S.lufengia* (14,647 bp), *S.mazbii* (14,496 bp), and *S.multidens* (14,590 bp) were similar, while *S.calcarata* had the longest mitogenome at 15,458 bp (Fig. 1). This sequence length is a common characteristic within Scolopendromorpha.

The six mitogenomes displayed the typical gene composition found in most eumetazoans, including 13 PCGs, two rRNAs, and one CR. In this study, all six species show varying degrees of tRNA loss, with the number of missing tRNAs ranging from two (*S.cataracta* lacked *trnC* and *trnL2*) to four (*S.alcyona* lacked *trnY*, *trnR*, *trnE*, and *trnL2*; *S.calcarata* lacked *trnD*, *trnR*, *trnE*, and *trnL2*; and *S.multidens* lacked *trnD*, *trnR*, *trnE*, and *trnL2*) (Fig. 1, Suppl. material 3).

**Figure 2.**
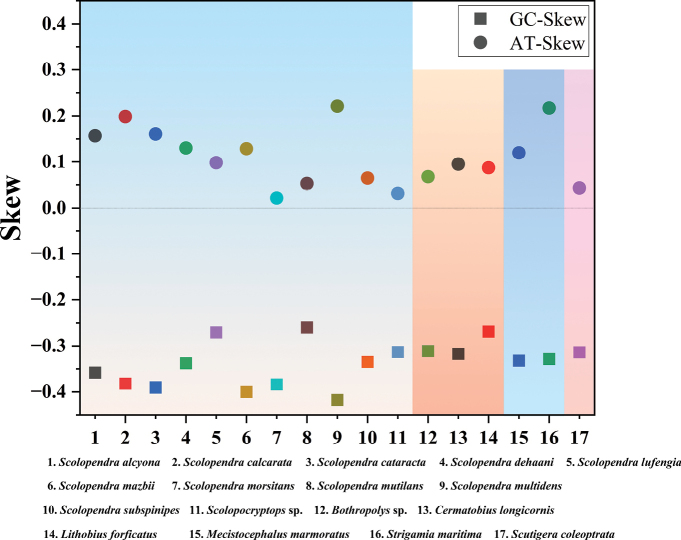
AT-skew and GC-skew of 17 Chilopoda species.

The nucleotide composition of the six newly sequenced genomes exhibited a strong bias towards A and T, as shown in Fig. 2. The AT-skew values were slightly positive, whereas the GC-skew values were significantly negative, ranging from −0.270 to −0.417 (Fig. 2). The analysis revealed a clear preference for C and a certain preference for A across the entire genomes. Additionally, PCGs, rRNAs, tRNAs, and CRs all exhibited a nucleotide composition bias with a higher percentage of ([A + T]%) compared to ([G + C]%) (Fig. 2).

### ﻿Protein-coding genes and codon usage

In the six mitogenomes, most PCGs (*cox1*, *cox2*, *cox3*, *atp6*, *atp8*, *nad2*, *nad3*, *nad6*, *cob*) were located on the majority strand, with four genes (*nad1*, *nad4*, *nad4l*, and *nad5*) encoded on the minority strand (Fig. 1).

The total lengths of the PCGs in *S.alcyona*, *S.calcarata*, *S.cataracta*, *S.lufengia*, *S.mazbii*, and *S.multidens* were 10,870 bp, 10,893 bp, 10,872 bp, 10,878 bp, 10,884 bp, and 10,848 bp, respectively, accounting for 75.13%, 70.29%, 74.16%, 73.99%, 74.60%, and 74.11% of the total mitogenome length (Table 2). The variations in these percentages are due to the differing lengths of the CR.

**Table 2.** Skewness of AT content, GC content, and genome-wide analysis of PCGs, tRNAs, rRNAs, and CRs in the six species investigated in this study.

Species	Region	Length (bp)	T (%)	C (%)	A (%)	G (%)	A+T (%)	C+G (%)	GC-Skew	AT-Skew
*Scolopendraalcyona*	Total genome	14,422	30.53	18.74	41.87	8.860	72.40	27.60	-0.357	0.156
	PCGs	10,870	39.70	14.64	31.67	14.00	71.36	28.64	-0.022	-0.112
	tRNAs	1107	37.43	11.08	38.72	12.77	76.15	23.85	0.070	0.016
	rRNAs	1900	7.06	43.38	31.60	17.96	74.99	25.01	-0.414	0.327
	CR	542	41.02	13.84	33.77	11.37	74.79	25.21	-0.097	-0.096
*Scolopendracalcarata*	Total genome	15,458	31.15	19.96	40.34	8.55	71.49	28.51	-0.400	0.128
	PCGs	10,893	38.60	16.22	30.89	14.29	69.49	30.51	-0.063	-0.110
	tRNAs	1145	36.86	11.11	38.57	13.46	75.43	24.57	0.095	0.022
	rRNAs	1886	42.68	7.45	30.92	18.95	73.60	26.40	0.435	-0.159
	CR	818	37.23	11.88	42.60	8.290	79.83	20.17	-0.177	0.067
*Scolopendracataracta*	Total genome	14,596	33.62	16.18	40.92	9.290	74.53	25.47	-0.270	0.097
	PCGs	10,872	41.45	12.83	32.37	13.35	73.82	26.18	0.019	-0.123
	tRNAs	1223	38.34	9.98	38.24	13.44	76.58	23.42	0.147	-0.001
	rRNAs	1904	41.60	7.49	35.30	15.61	76.90	23.10	0.351	-0.081
	CR	813	39.27	11.96	34.98	13.78	74.25	25.75	0.070	-0.057
*Scolopendralufengia*	Total genome	14,647	27.87	21.02	41.71	9.40	69.58	30.42	-0.381	0.198
	PCGs	10,878	38.56	16.29	29.75	15.41	68.31	31.69	-0.027	-0.128
	tRNAs	1089	36.31	11.96	37.99	13.75	74.29	25.71	0.069	0.022
	rRNAs	1933	43.88	8.53	28.09	19.51	71.97	28.03	0.391	-0.219
	CR	656	34.67	15.83	39.35	10.14	74.02	25.98	-0.219	0.063
*Scolopendramazbii*	Total genome	14,496	28.12	22.95	38.87	10.05	67.00	33	-0.390	0.160
	PCGs	10,884	36.58	18.67	28.67	16.08	65.25	34.75	-0.074	-0.121
	tRNAs	1142	35.32	12.73	38.65	13.30	73.97	26.03	0.021	0.045
	rRNAs	1940	41.60	8.22	29.40	20.78	71.01	28.99	0.433	-0.171
	CR	584	35.09	14.06	37.56	13.29	72.64	27.36	-0.028	0.034
*Scolopendramultidens*	Total genome	14,590	26.98	21.76	42.32	8.94	69.29	30.71	-0.417	0.221
	PCGs	10,848	37.26	16.81	30.79	15.13	68.05	31.95	-0.052	-0.095
	tRNAs	1175	36.10	12.28	37.30	14.31	73.41	26.59	0.076	0.016
	rRNAs	1937	43.69	8.04	28.11	20.16	71.80	28.2	0.429	-0.216
	CR	645	38.67	14.57	35.64	11.11	74.31	25.69	-0.134	-0.040

Most of the PCGs in these mitogenomes initiated with the standard start codons ATG, ATT, or ATA, except for two species (*S.calcarata*, *S.mazbii*), where ATC was used as the initiation codon in the *nad3* gene. Notably, the start codon of the *cox1* gene in all six species was CGA, matching that of the reference species *S.dehaani* (KY947341.1). None of the species examined in this study contained any unusual stop codons; the typical stop codons TAA, TAG, and incomplete codons represented as TA- and T--.

**Figure 3.**
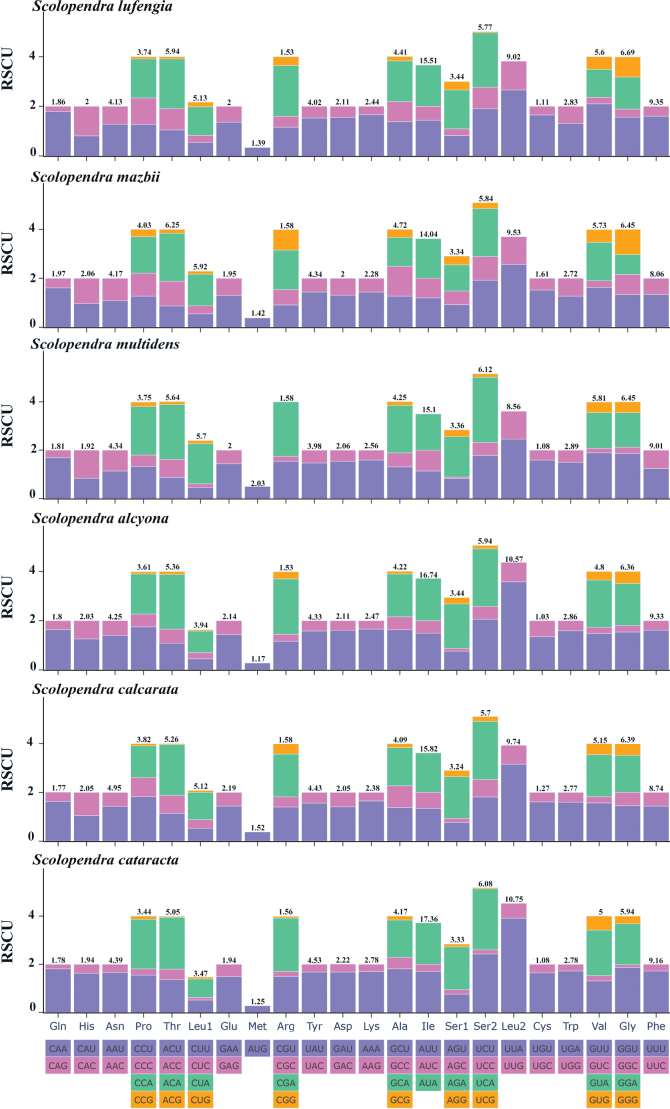
RSCU of six *Scolopendra* species. Stop codons are excluded.

The comparative analysis of Relative Synonymous Codon Usage (RSCU) values across all PCGs revealed that the codon usage patterns of these six species were similar (Fig. 3). Codons encoding Ile and Leu2 were used frequently, while codons for Cys and Met were less common. The most frequently used codons were composed of A or C, while those less frequently used contained T or G. This pattern indicated that the RSCU values are positively correlated with the AT bias in the PCGs.

The results showed that the Ka/Ks ratios for all examined PCGs were < 1, suggesting that these genes have undergone purifying selection throughout their evolution (Sun et al. 2024). Among the 13 PCGs, the *atp8* gene exhibited the highest Ka/Ks ratio, indicating greater amino acid diversity and a faster evolutionary rate, likely due to lower selection pressure. In contrast, the *cox1* gene had the lowest Ka/Ks ratio, reflecting slower evolution and stronger selective constraint (Suppl. material 1).

In addition to the Ka/Ks ratio analysis, genetic divergence between Chilopoda species was further assessed by calculating the cumulative p-distance across the nucleotide sequences of the 13 PCGs. The p-distance results confirmed that the *atp8* gene was the fastest evolving, consistent with the Ka/Ks analysis (Suppl. material 2).

### ﻿Ribosomal and transfer RNA genes and control regions

The size of the *rrnL* gene ranged from 1147 bp (*S.lufengia*) to 1215 bp (*S.mazbii*), while the size of the *rrnS* gene varied between 708 bp (*S.calcarata*) and 744 bp (*S.alcyona*). Both rRNA genes were located between *trnL1* or *trnL2* and *trnI*, with *trnV* separating them. The sizes of the tRNA genes ranged from 38 bp (*trnT* in *S.cataracta*) to 79 bp (*trnP* in *S.alcyona*).

Mitogenomes exhibit two distinct non-coding sequences: the control region (CR) and the intergenic spacers (IGs). The CRs varied in size, ranging from 542 bp (*S.alcyona*) to 818 bp (*S.calcarata*). *S.multidens* and *S.lufengia* had an IGs, measuring 83 bp, 273 bp, respectively. Additionally, *S.calcarata* contained an extra CR with a length of 867 bp, and its A+T content was as high as 79.95%. The variation in mitogenome size between the centipede species is largely attributed to differences in CR length, with the particularly long sequence in *S.calcarata* likely due to its large-scale CR.

The A+T content of the CRs in these six mitogenomes varied: 74.79% (*S.alcyona*), 79.83% (*S.calcarata*), 74.25% (*S.cataracta*), 74.02% (*S.lufengia*), 72.64% (*S.mazbii*), and 74.31% (*S.multidens*).

### ﻿Gene rearrangement

**Figure 4.**
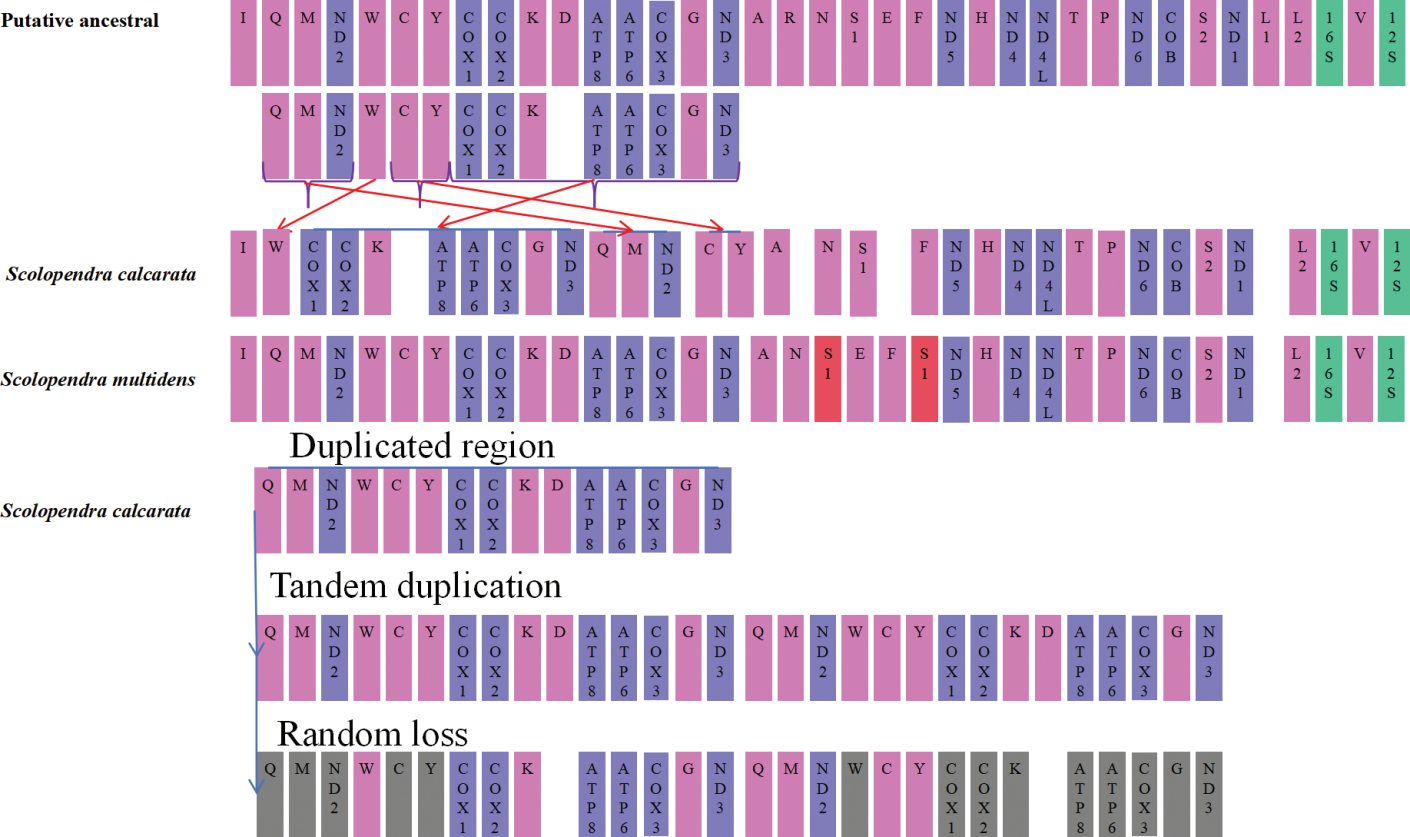
Hypothesized ancestral gene arrangement of the Scutigeromorpha order. Red arrows indicate recombination events of *S.calcarata*, and red rectangles represent duplicated genes of *S.multidens*. The hypothetical gene rearrangement process via the tandem duplication/random loss model is depicted schematically, with shaded areas indicating partially deleted genes or regions.

The mitogenome arrangements in *S.alcyona*, *S.cataracta*, *S.lufengia*, and *S.mazbii* are consistent with the inferred ancestral arrangement in Myriapoda (Wang et al. 2022). In *S.multidens*, the *trnS1* gene experienced a tRNA duplication event, leading to the formation of an identical fragment (Fig. 4). The gene order in *S.calcarata* has rearranged from the ancestral configuration (*trnQ*-*trnM*-*nad2*-*trnW*-*trnC*-*trnY*-*cox1*-*cox2*-*trnK*-*atp8*-*atp6*-*cox3*-*trnG*-*nad3*) to a derived arrangement (*trnW*-*cox1*-*cox2*-*trnK*-*atp8*-*atp6*-*cox3*-*trnG*-*nad3*-*trnQ*-*trnM*-*nad2*-*trnC*-*trnY*) (Fig. 4). This suggests that *S.multidens* has undergone a single tRNA duplication event, while *S.calcarata* has experienced a rare gene order rearrangement event involving PCGs.

### ﻿Phylogenetic analysis

**Figure 5.**
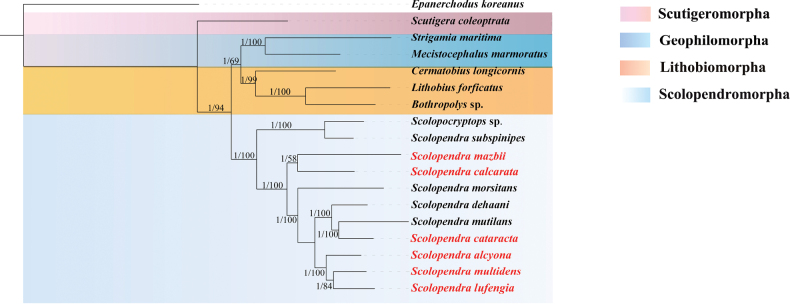
Phylogenetic tree based on 13 PCGs of 17 Chilopoda species and one outgroup. Numbers at the nodes represent posterior probabilities and bootstrap values from BI and ML analysis, respectively. The novel species introduced in this manuscript are marked in red.

Both BI and ML analyses produced identical topologies. As illustrated in Fig. 5, Scutigeromorpha represents the most basal lineage within Chilopoda. Geophilomorpha and Lithobiomorpha form a distinct clade, suggesting a sister-group relationship between these two taxa (Fig. 5).

In the phylogenetic tree constructed based on 13 PCGs, *S.alcyona*, *S.dehaani*, *S.mutilans* L. Koch, 1878, *S.cataracta*, *S.lufengia*, *S.morsitans* Linnaeus, 1758, and *S.multidens* form a highly supported monophyletic clade (BI posterior probability = 1, ML bootstrap value = 100). Within this clade, *S.lufengia* and *S.multidens* first form a sister group, which then clusters with *S.alcyona* to constitute a subclade. This subclade ultimately becomes sister to the clade comprising *S.cataracta*, *S.mutilans* and *S.dehaani* (Fig. 5).

Conversely, *S.mazbii* and *S.calcarata* cluster into an independent clade (BI posterior probability = 1, ML bootstrap value = 100), which diverges significantly from the clades of other *Scolopendra* species (Fig. 5). This suggests that the two species may have unique evolutionary trajectories. The topological structure indicates that there are two major evolutionary lineages within *Scolopendra*, whose differentiation patterns show a certain correlation with gene rearrangement events, such as the large-scale gene rearrangement in *S.calcarata* and the tRNA duplication in *S.multidens*. *Scolopendrasubspinipes* clusters with *Scolopocryptops* sp. on the same branch, a result differing from previous studies and suggesting that the genus *Scolopendra* may not be monophyletic (Fig. 5).

## ﻿Discussion

The observed tandem duplication events in the mitochondrial genome of *S.multidens* provide potential evidence supporting the TDRL model (Jiang et al. 2016). Two models of TDRL and recombination are generally proposed to explain this rearrangement mechanism. It is plausible to hypothesize that the gene arrangement pattern with an extra *trnS1* gene could be explained through the TDRL model (Madsen et al. 1993; Hassanin 2006). In this study, we propose that the formation of the *S.calcarata* mitogenome can be explained by the TDRL model and the slipped-strand mispairing mechanism (Madsen et al. 1993; Hassanin 2006). Based on molecular mechanisms, the mitochondrial DNA region (*trnQ*–*trnM*–*nad2*–*trnC*–*cox1*–*cox2*–*trnK*–*trnD*–*atp8*–*atp6*–*cox3*–*trnG*–*nad3*) in *S.calcarata* first underwent tandem duplication, followed by random loss of partial genes (Fig. 2). Our analysis is limited by the small sample size of mitogenomes; future studies with broader taxonomic sampling may refine these phylogenetic relationships. Therefore, more cases of mitochondrial gene rearrangements are needed to further elucidate the underlying mechanisms.

The most extensive overlap was observed between *trnI* and *trnQ*, where a 30 bp overlap was present in six species. In arthropods, the independent loss of individual tRNA gene segments is common, and overlaps between tRNAs on different strands are widely conserved (Jühling et al. 2012). Truncations were observed in certain tRNAs, with some retaining only a single stem-loop structure. Furthermore, the tRNAs cannot be identified or predicted, and their secondary structures cannot be predicted using the tRNAscan-SE Search Server or rtools (Jühling et al. 2012; Xue et al. 2016; Ding et al. 2022). In *S.multidens* and *S.lufengia*, each species harbors a unique non-coding genomic region that shows no sequence similarity to other genomic segments. These regions may represent either putative CRs or transitional pseudogene evolution. And, *S.calcarata* had an extra CR. Mitochondrial DNA tandem duplication initially generates a duplicated region, replicating both structural genes and extra transcriptional regulators in non-coding regions. Errors in this process may lead to the retention of duplicated transcriptional regulators (Lavrov et al. 2002). The recurrence of the CR is likely a result of concerted evolution of the duplicated control regions (Kumazawa et al. 1998).

Gene rearrangements can significantly influence gene expression and genome replication, contributing to genomic variation, molecular adaptation, and various physiological and life-history traits (Gissi et al. 2008). Additionally, this study further validated the relationship between *S.multidens* and *S.mutilans* (Hu et al. 2020) using complete mitogenomes. *Scolopendramultidens* and *S.mutilans* had previously been considered the same species due to their highly similar appearances (Vahtera and Edgecombe 2014), but were found to be distantly related. *Scolopendrasubspinipes* Leach, 1815 clusters with *Scolopocryptops* sp. on the same branch, which is consistent with the findings of Wang et al. (2022). This result indicates that the monophyly of the genus *Scolopendra* is controversial, and its taxonomic status requires further investigation. Recent research suggests precipitation regulates genome size indirectly affecting gene rearrangement frequency (Zhang et al. 2024a), findings that warrant future ecological-genomic correlation analyses to elucidate *Scolopendra*’s adaptive evolution mechanisms.

In this study, data from some Scolopendromorpha species were excluded due to invalid annotation information in NCBI. We speculate that this is likely attributed to the unique tRNA characteristics of Scolopendromorpha species, which may have hindered the annotation system from accurately identifying their tRNA genes. To address this, future research should incorporate a broader range of *Scolopendra* species and chromosome-level genomic data to refine phylogenetic inference (Zhang et al. 2024b), validate the universality of gene rearrangement mechanisms across the genus, and provide more robust molecular evidence for taxonomic revisions within *Scolopendra*.

## ﻿Conclusion

The mitogenomes of *S.alcyona*, *S.cataracta*, *S.lufengia*, and *S.mazbii* show conserved arrangements consistent with the ancestral myriapod pattern, while *S.multidens* and *S.calcarata* exhibit derived tRNA duplication and gene rearrangement, respectively. Phylogenetic analysis reveals two distinct monophyletic clades within *Scolopendra*, with the placement of *S.subspinipes* alongside *Scolopocryptops* sp. challenging the genus’ monophyly. These findings suggest potential subgeneric classification for the two clades and warrant re-evaluation of *S.subspinipes*’s taxonomic status, highlighting the need for expanded sampling of global populations and integration of long-read sequencing and nuclear markers to refine phylogenetic inference and resolve taxonomic ambiguities in Scolopendromorpha.

## ﻿Additional information

### Use of AI

No use of AI was reported.

### Author ORCIDs

Mengcheng Wan https://orcid.org/0009-0005-8223-8016

Hongyi Liu https://orcid.org/0000-0003-2081-5779

Cuiqing Gao https://orcid.org/0000-0002-0177-5161

### Data availability

The data that support the findings of this study are openly available in National Center for Biotechnology Information at https://www.ncbi.nlm.nih.gov/nuccore, reference numbers PP816935, PP855996−PP856000.

## Supplementary materials

Supplementary material 1 Ka/Ks values for the 13 PCGs Data type jpg Explanation note Pale pink box plots represent ten species of Scolopendromorpha, while pale blue box plots represent seventeen species of Chilopoda. The band inside the box indicates the median, with the upper and lower hinges corresponding to the 25^th^ and 75^th^ percentiles. Circles represent outliers. https://binary.pensoft.net/file/1392135This dataset is made available under the Open Database License (http://opendatacommons.org/licenses/odbl/1.0/). The Open Database License (ODbL) is a license agreement intended to allow users to freely share, modify, and use this Dataset while maintaining this same freedom for others, provided that the original source and author(s) are credited.Mengcheng Wan, Yiwen Yang, Jiachen Wang, Gaoji Zhang, Hongyi Liu, Cuiqing Gao

Supplementary material 2 Genetic p-distances for nucleotide sequences of 13 PCGs and 2 rRNAs Data type jpg Explanation note Pale pink box plots represent ten species of Scolopendromorpha, while pale blue box plots represent seventeen species of Chilopoda. https://binary.pensoft.net/file/1392136This dataset is made available under the Open Database License (http://opendatacommons.org/licenses/odbl/1.0/). The Open Database License (ODbL) is a license agreement intended to allow users to freely share, modify, and use this Dataset while maintaining this same freedom for others, provided that the original source and author(s) are credited.Mengcheng Wan, Yiwen Yang, Jiachen Wang, Gaoji Zhang, Hongyi Liu, Cuiqing Gao

Supplementary material 3 Features of the mitogenomes of *S.alcyona*, *S.calcarata*, *S.cataracta*, *S.lufengia*, *S.mazbii*, and *S.multidens* Data type xlsx https://binary.pensoft.net/file/1392137This dataset is made available under the Open Database License (http://opendatacommons.org/licenses/odbl/1.0/). The Open Database License (ODbL) is a license agreement intended to allow users to freely share, modify, and use this Dataset while maintaining this same freedom for others, provided that the original source and author(s) are credited.Mengcheng Wan, Yiwen Yang, Jiachen Wang, Gaoji Zhang, Hongyi Liu, Cuiqing Gao
