# Supplementary figures and images for: ﻿Comparative analysis of mitogenomes in six Scolopendra species (Chilopoda, Scolopendromorpha): insights into rare genetic rearrangements and phylogeny
Source: Zookeys. 2025 Aug 11;1248:341–58. doi: 10.3897/zookeys.1248.159578 (PMC12361922; doi:10.3897/zookeys.1248.159578)

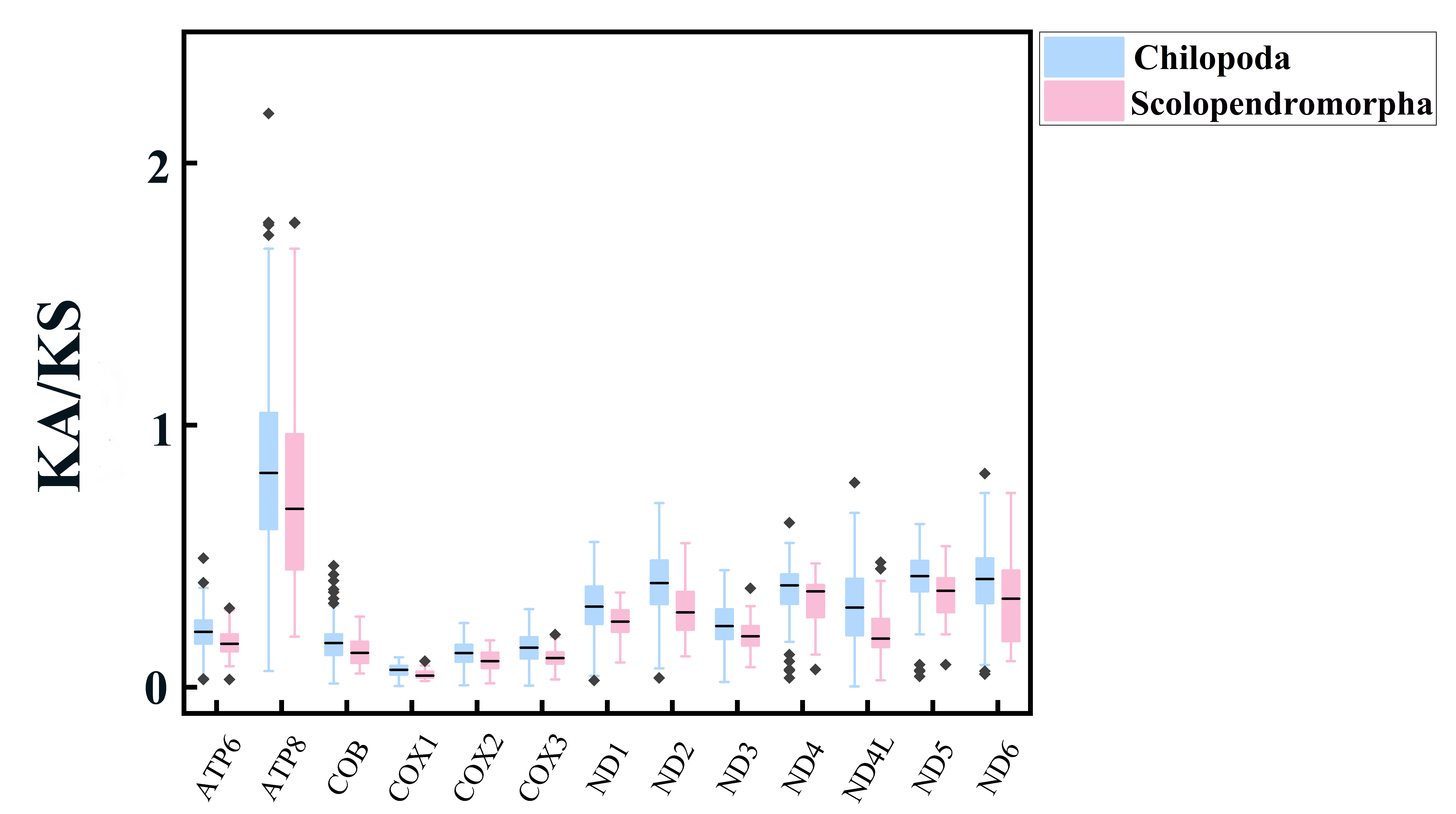

Supplement: Supplementary material 1 — Ka/Ks values for the 13 PCGs [file zookeys-1248-341_article-159578__-s001.jpg]

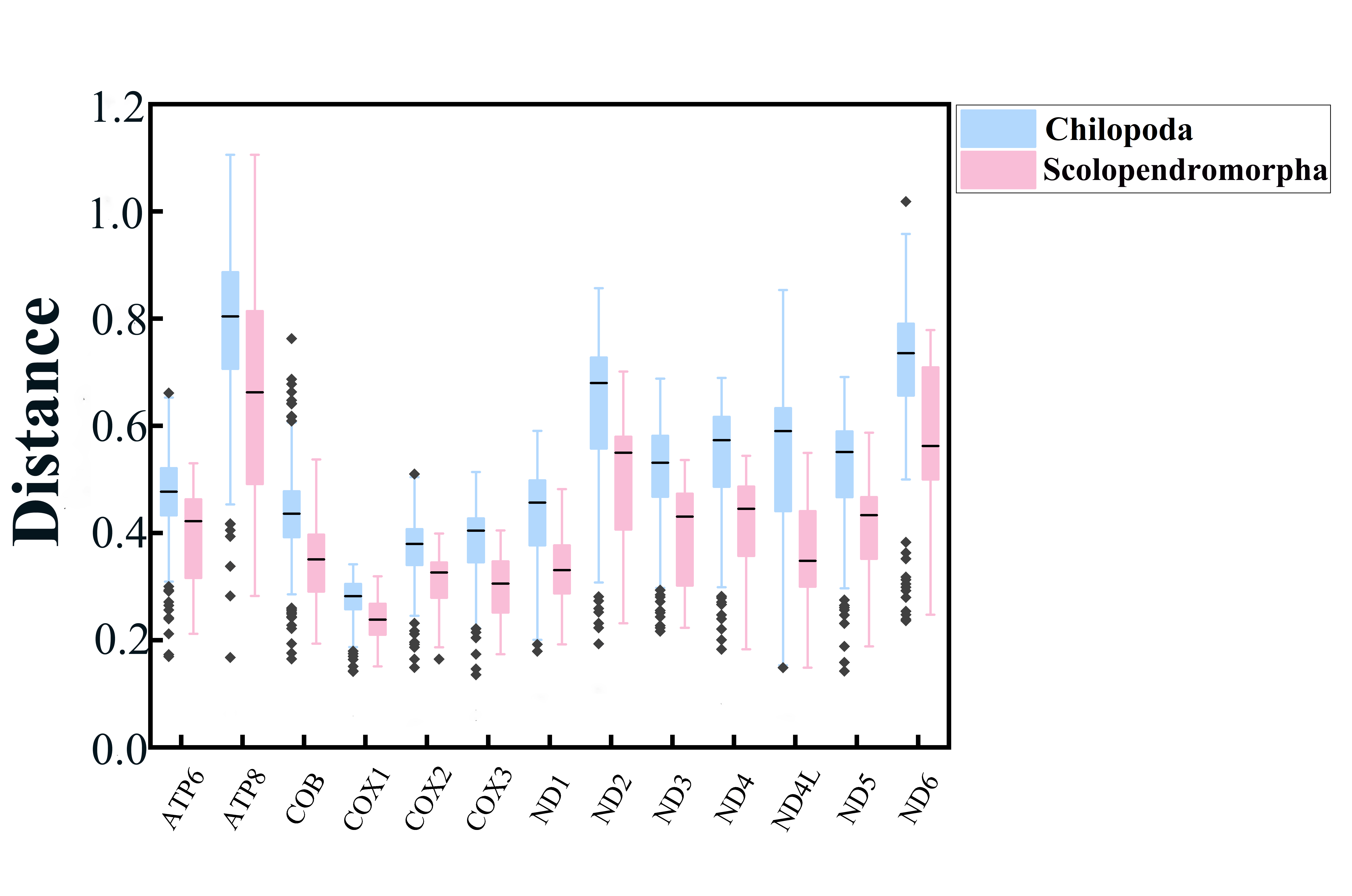

Supplement: Supplementary material 2 — Genetic p-distances for nucleotide sequences of 13 PCGs and 2 rRNAs [file zookeys-1248-341_article-159578__-s002.jpg]
